# Relationships between depressive symptoms and self-reported unintentional injuries: the cross-sectional population–based FIN-D2D survey

**DOI:** 10.1186/1471-2458-12-516

**Published:** 2012-07-10

**Authors:** Katariina Korniloff, Arja Häkkinen, Hannu J Koponen, Hannu Kautiainen, Salme Järvenpää, Markku Peltonen, Pekka Mäntyselkä, Olli Kampman, Heikki Oksa, Mauno Vanhala

**Affiliations:** 1 Department of Health Sciences, University of Jyväskylä, PL 35, Jyväskylä FI-40014, Finland; 2 Department of Physical and Rehabilitation Medicine, Central Finland Central Hospital, Keskussairaalantie 19, FI-40620, Jyväskylä, Finland; 3 Department of Psychiatry, Kuopio University Hospital, Kuopio, Finaland; 4 University of Eastern Finland, Kuopio, FI-70210, Finland; 5 Unit of Family Practice, Central Finland Central Hospital, Keskussairaalantie 19, FI-40620, Jyväskylä, Finland; 6 Unit of Primary Health Care, Kuopio University Hospital, PO Box 1777, Kuopio FI-70211, Finland; 7 Medcare foundation, Jyväskylä, Finland; 8 Chronic Disease Epidemiology and Prevention Unit, National Institute for Health and Welfare, P.O. Box 30, Helsinki FI-00271, Finland; 9 Unit of Primary Health Care, University of Eastern Finland, PO Box 1627, Kuopio Campus FI-70211, Finland; 10 Department of Psychiatry, Seinäjoki Hospital District and Medical School, University of Tampere, Tampere FI-33014, Finland; 11 Tampere University Hospital, Teiskontie 35, Tampere, FI-33521, Finland

**Keywords:** Beck depression inventory, Depressive symptoms, Injury, Physical activity

## Abstract

**Background:**

There is a lack of knowledge on the influence of different levels of physical activity (PA) on unintentional injuries among those with depressive symptoms (DS). The aim of this study was to evaluate the relationship between PA categories and unintentional injuries among participants with and without DS based on a cross-sectional population–based FIN-D2D survey conducted in 2007.

**Methods:**

Out of 4500, 2682 participants (60%) aged 45–74 years attended in this study. The unintentional injuries over the past year were captured in a questionnaire. DS were determined with the Beck Depression Inventory (≥ 10 points) and PA with the International Physical Activity Questionnaire. The statistical significance between DS and unintentional injury categories was evaluated by using *t*-test, chi-square test, or permutation test, analysis of covariance, or regression models. The factors related to unintentional injuries were estimated by univariate and multivariate logistic regression models.

**Results:**

The proportion of subjects with unintentional injuries was higher among those with DS (17%) compared to those without DS (10%) (age- and gender-adjusted *p* = 0.023). The median (range) number of activity-loss days after injury was 22 (0–365) in participants with DS and 7 (0–120) in participants without DS ( *p* = 0.009). The percentage of subjects with unintentional injuries was not significantly different between PA categories in participants with DS and without DS. A stepwise multivariate logistic regression analysis showed that DS, functional ability, and musculoskeletal diseases were related to unintentional injuries.

**Conclusions:**

PA level was not related to unintentional injuries, whereas those with DS had a higher prevalence of unintentional injuries and prolonged activity-loss after injury. These results underline the importance of injury prevention, especially among those who have DS and additional risk factors.

## Background

A sedentary lifestyle is commonly associated with several deleterious health consequences, one of which is depressive symptoms (DS). A number of studies have found DS to be associated with unintentional injury [1-4]. In fact, DS have proved to increase the risk of injury by 41% [4]. It is also evident that the risk of DS increases due to unintentional injury [5]. Hence, this phenomenon seems to be bidirectional as demonstrated in a national representative study [2].

Further several socio-demographic factors can be associated with unintentional injuries. These high-risk characteristics are male gender, low income, prior injury, and insufficient night’s sleep [2,4,6]. Additionally, children, older people, and those who are less educated and have fewer social resources are at highest risk for injuries [7]. Furthermore, an estimated 40–60% of injuries in Europe may be associated with alcohol consumption [7]. Therefore, binge drinking habits among Finns might be remarkable in terms of unintentional injuries [8].

Moreover, behavioral factors like a physically active lifestyle also play a role in the phenomenon of unintentional injuries. Increased physical activity (PA), particularly increased strength and balance training, has proved beneficial in terms of decreasing falling injuries and preventing fractures [9]. Despite several health benefits of PA [10], it may also have some deleterious consequences. Hootman et al. observed that an increased duration of PA per week and a higher cardiorespiratory fitness level were associated with an increased risk of activity-related injury, and that moderate types and duration of PA have lower injury risks than more vigorous types and longer durations of PA [11]. However, a consistent trend was evident when a sedentary lifestyle was compared with a physically active one, with the latter providing some protection against injuries other than activity-related ones [11].

To our knowledge, there is no research concerning the influence of different levels of total PA on unintentional injuries among those with DS. However, the level of PA could decrease as a result of DS. This tendency may then decrease the incidence of activity-related injuries. On the other hand, a sedentary lifestyle is associated with worse functional ability [12,13], which may in turn increase the prevalence of injuries. Thus, the aim of the present cross-sectional population-based study was to describe the relationship between total PA and unintentional injuries among participants with and without DS. An additional aim was to evaluate the prevalence of unintentional injuries in relation to sociodemographic and clinical characteristics as well as functional ability.

## Methods

The Finnish type 2 diabetes (FIN-D2D) population survey was carried out in the hospital districts of Pirkanmaa, Southern Ostrobothnia and Central Finland, between October and December 2007 [14,15]. A random sample of 4500 subjects aged 45–74 years, stratified according to gender, 10-year age groups (45–54, 55–64 and 65–74 years), and the three geographical areas was selected from the National Population Register in August 2007. The study participants were invited by mail to a clinical examination. Of the 4500 persons solicited, 2868 participated in the health examination (attendance 64%). Non-responders were on average 1.4 years younger than responders (*p* < 0.001), and female were more likely to participate than male (67% vs. 61%, *p* < 0.001). After exclusion of 90 subjects because of incomplete information about physical activity, injuries, or DS, the final study group included 2682 (60%) persons (53% females) with a mean (SD) age of 59 (8) years. All participants signed an informed consent form. Ethical permission for the study was granted by the Hospital District of Helsinki and the Uusimaa ethics committee.

The health examination methods followed the multinational monitoring of trends and determinants in cardiovascular disease (MONICA) protocol [16], and were made by nurses specially trained in the survey procedures. Height and weight were measured to calculate body mass index (BMI). All participants completed a standard questionnaire including questions about years of education, medications, alcohol use, smoking, physical function, physical activity, and injuries. Information about the participants’ chronic diseases during the past 12 months was also captured with questionnaire and categorized into groups of cardiovascular, respiratory, and musculoskeletal diseases.

Alcohol-specific questions inquired about the frequency of times during the past 12 months of drinking alcohol to feel inebriated. Alcohol consumption was classified into four categories as follows: never (0), occasionally (1), at least once a month (2), and at least once a week (3).

Functional ability was evaluated with the modified functional ability questionnaire including questions about dressing, cutting toenails, climbing stairs, walking, carrying, running, cycle and travelling [17-20]. Answers (1, able without difficulties; 2, able but is difficult; 3, cannot) were summed in a total score, which accounted for the physical functioning index. For the analysis, the total score was standardized into the scale of 0–10 with higher numbers indicating worse functioning.

Physical activity (PA) was assessed with the self-administered short version of the International Physical Activity Questionnaire (IPAQ). This questionnaire estimates the frequency, intensity, and duration of PA during leisure times, at home, and also occupational and commuting physical activity over the previous seven days [21-24]. Total daily PA in MET-min (which is Metabolic Equivalent; 1 MET = resting energy expenditure) was calculated according to the official IPAQ scoring protocol ( http://www.ipaq.ki.se). In the analysis, PA was divided into three categories (low < 600, moderate 600–2999, and high ≥ 3000) based on MET-min week^-^¹.

The questionnaire asked about unintentional injuries which required medical attention during the past 12 months, and divided them into the following four groups: traffic injuries, home injuries, sports or exercise injuries, and other leisure-time injuries. The questionnaire also asked about the number of activity-loss days, which refers to difficulties coping with daily activities.

DS were assessed with the Beck Depression Inventory (BDI) [25]. The BDI is a 21-item self-report questionnaire consisting of symptoms and attitudes related to depression. The items are summed in a total score that ranges from 0 to 63; higher numbers indicate greater depression. The cut–off point for DS was 10, which indicates at least mild depression [26,27]. Participants with DS ≥ 10 were offered the possibility of meeting with a psychiatrist for further investigation.

### Statistical analysis

The data are presented as means with standard deviations (SD) or as counts with percentages. The most important outcomes are given with 95% confidence intervals (CI). The comparison between groups was made using *t*-test, chi-square test, or permutation test. When adjusting for confounding factors, analysis of covariance, logistic regression model, or ordered logistic regression model was applied. Univariate logistic regression models and multivariate logistic regression model with forward stepwise selection (probability for entry 0.05) were used to investigate factors related to unintentional injuries. Visual assessment of residuals and influence diagnostics were used in validating the assumptions underlying the logistic regression model.

## Results

Three hundred one participants reported at least one unintentional injury during the past 12 months. Table 1 shows the demographic and clinical factors according to unintentional injury status. Participants with unintentional injuries were older, had higher BMI value, higher prevalence of DS, worse functional ability, higher prevalence of cardiovascular, respiratory and musculoskeletal diseases, and greater alcohol use, compared with participants without unintentional injuries.

**Table 1:** Demographic and clinical data according to unintentional injuries

	**No unintentional injuries**	**Unintentional injuries**	***p*-value**^**a**^
	**N = 2381**	**N = 301**	
Demographic			
Female, n (%)	1260 (53)	153 (51)	0.49
Age, mean (SD)	59.3 (8.3)	60.4 (8.2)	0.034
Education years, mean (SD)	11.4 (3.6)	11.5 (4.2)	0.34
Body mass index, mean (SD)	27.4 (4.8)	38.0 (5.5)	0.050
Clinical			
Depressive symptoms present, n (%)	326 (14)	67 (22)	<0.001
Antidepressants, n (%)	129 (5)	20 (7)	0.34
Functional ability, mean (SD)	0.85 (1.33)	1.26 (1.77)	<0.001
Cardiovascular diseases, n (%)	820 (35)	132 (45)	0.005
Respiratory diseases, n (%)	187 (8)	36 (12)	0.026
Musculoskeletal diseases, n (%)	698 (30)	121 (41)	<0.001
Current smoking, n (%)	529 (22)	67 (22)	0.69
Alcohol use, n (%)			0.049
Never	1112 (47)	128 (43)	
Occasionally	841 (35)	115 (38)	
At least once a month	295 (12)	39 (13)	
At least once a week	133 (6)	19 (6)	
Physical activity category (MET min week^-1^), n (%)			0.28
Low (< 600)	410 (17)	47 (16)	
Moderate (600–2999)	1019 (43)	124 (41)	
High (≥ 3000)	952 (40)	130 (43)	

a: Adjusted for age and gender (except gender and age).

**Figure 1:**
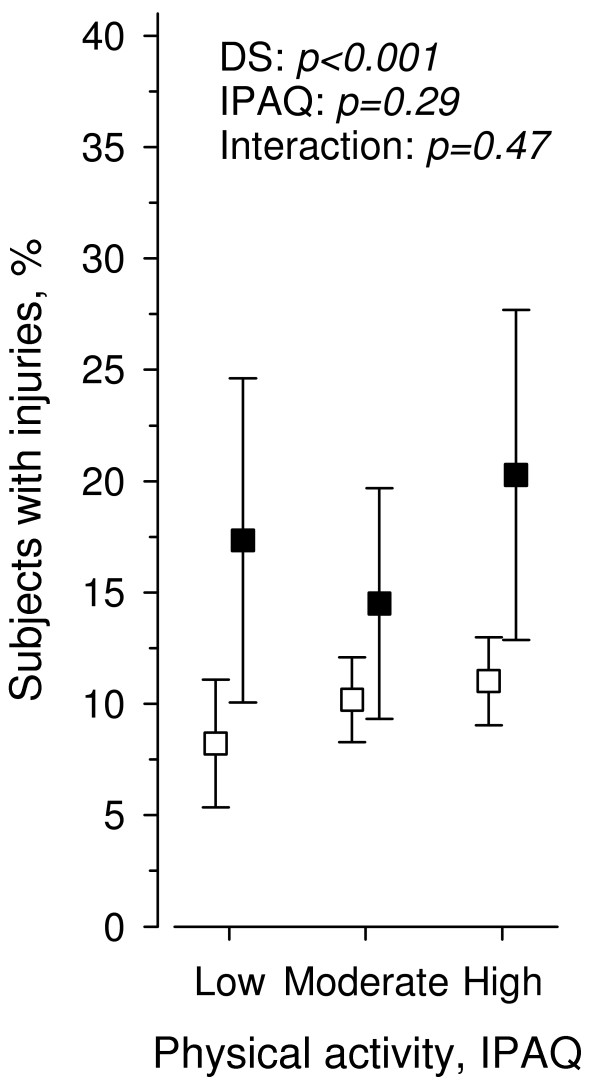
**Age- and gender-adjusted percentage of subjects with unintentional injuries in physical activity categories according to depressive symptoms (DS).** Percentages with 95% confidence intervals in participants with (black square) or without (open square) depressive symptoms are shown.

The prevalence of subjects with unintentional injuries among participants with DS was 17.0% (95% CI: 13.5% to 21.1%) and 10.2% (95% CI: 9.0% to 11.5%) among participants without DS (age- and gender-adjusted *p* < 0.001). The prevalence of subjects with unintentional injuries among those with low total PA was 10.3% (95% CI: 7.7% to 13.4%), among those with moderate PA 10.8% (95% CI: 9.1% to 12.8%), and high PA 12.0% (95% CI: 10.1% to 14.1%) (age- and sex adjusted *p* = 0.55). Figure 1 shows main effects and interaction of total PA and DS on unintentional injuries.

A home injury was the most commonly reported injury in both the DS present and not present groups, whereas other leisure time injury was second (Table 2). The mean (range) activity-loss days after injury was 22 (0–365) days in participants with DS and 7 (0–120) days in participants without DS (*p* = 0.009).

**Table 2:** Number of subjects with different causes of unintentional injuries according to depressive symptoms (DS)

	**DSn (%)**	**No DSn (%)**
Cause of injury		
Traffic injury	12 (15)	32 (12)
Home injury	33 (42)	104 (39)
Sports injury	8 (10)	50 (19)
Other leisure-time injury	26 (33)	81 (30)

Univariate logistic regression analysis showed that age, BMI, DS, functional ability, and cardiovascular, respiratory and musculoskeletal diseases were significantly related to a higher risk of injuries, but PA level was not. In the forward stepwise multivariate model, the relationship between unintentional injuries and DS, functional ability, and musculoskeletal diseases remained significant (Table 3).

**Table 3:** Univariate and multivariate stepwise logistic regression models for unintentional injuries

	**Univariate**		**Multivariate**^**a**^	
	**OR (95% CI)**	***p*-value**	**OR (95% CI)**	***p*-value**
Age	1.02 (1.00 to 1.03)	0.034		
Male gender	1.09 (0.86 to 1.38)	0.49		
Body mass index	1.03 (1.00 to 1.05)	0.032		
Education years	1.00 (0.97 to 1.03)	0.93		
Depressive symptoms	1.80 (1.34 to 2.43)	<0.001	1.52 (1.11 to 2.10)	0.010
Use of antidepressants	1.24 (0.76 to 2.02)	0.38		
Functional ability	2.31 (1.62 to 3.30)	<0.001	1.74 (1.15 to 2.61)	0.008
Cardiovascular diseases	1.50 (1.18 to 1.92)	<0.001		
Respiratory diseases	1.59 (1.09 to 2.33)	0.016		
Musculoskeletal diseases	1.63 (1.27 to 2.08)	<0.001	1.38 (1.06 to 1.80)	0.018
IPAQ		0.27^b^		
< 600	1 (reference)			
600–2999	1.06 (0.74 to 1.51)			
≥ 3000	1.19 (0.84 to 1.70)			
Alcohol use		0.24^b^		
Never	1 (reference)			
Occasionally	1.19 (0.91 to 1.55)			
At least once a month	1.15 (0.78 to 1.68)			
At least once a week	1.24 (0.74 to 2.08)			

OR, odds ration; CI, confidence interval; IPAQ, International Physical Activity Questionnaire.

a: Forward stepwise selection. Out of all variables used in univariate model only those are shown which remined stististically significant. (probability for entry 0.05).

b: p for linearity.

## Discussion

This population-based survey showed that depressive symptoms (DS) were strongly related to unintentional injuries and that the proportion of subjects with unintentional injuries was almost double among those with DS compared with those without DS. This inequality becomes even more relevant when taking into account that the number of activity-loss days after injury in participants with DS proved to be threefold that of those without DS in this study. However, contrary to our expectation, physical activity level was not related to unintentional injuries among those with or without DS. Since injury prevention has become an important focus of government policy in Finland, it would be worthwhile to draw attention to the various factors behind home and other leisure-time unintentional injuries, which were the most common injuries identified in this study.

Reasons behind the relationship between DS and unintentional injuries can be speculated. In the analysis variety of known confounders was controlled including antidepressant use. Most of the previous studies examining injuries and mental health have reported antidepressant medication to be a risk factor for injuries [28,29], while the results of this study were not similar. In addition, Tiesman et al. found that DS are a risk factor for injury, regardless of the use of antidepressants [4]. Hence, some alternative explanations for the relationship may be found which have, however, not been examined in this study. Perhaps increased daytime sleepiness and a lack of concentration among those with DS may act as connecting factors [30].

Moreover, some differences in clinical factors were found between participants with and without unintentional injuries in addition to DS. Those with unintentional injuries reported higher prevalence of somatic diseases and worse functional ability as well. These factors reached statistical significance in univariate analysis, and functional ability and musculoskeletal diseases were related to unintentional injuries in multivariate analysis as well. Functional ability worsens because of musculoskeletal diseases [31] and likely due to other somatic diseases as well. Therefore improving functioning could have positive impact on injury prevention. Improvement in functioning might be remarkable with respect to injury prevention planning and primary care settings [32].

In the univariate analysis, older age and higher BMI proved to be significant demographic factors related to unintentional injuries, as found in earlier research [7,33]. However, in contrast to previous studies male gender, years of education, and alcohol use did not seem related to the unintentional injury rate in this population-based study [2,4,7]. Reasons behind why alcohol use was not related to unintentional injuries can be speculated. Firstly, the participation rate of heavy drinkers was likely to have been low and, furthermore, the respondents may have underestimated their alcohol consumption to provide socially desirable answers.

The most common injuries appeared to be home and other leisure-time unintentional injuries. This finding is in line with a previous report from Finland [34]. Sports and traffic unintentional injuries seemed to play a minor role in the present population-based study. We found that only a minority of all unintentional injuries were sports-related both among those with DS (10%) and without DS (19%). Sports-related injuries may be more likely to occur among physically active persons [35]. This probably explains the difference in the prevalence of sports-related injuries between those with and without DS.

Most of the earlier studies about physical activity as an underlying factor in injuries have focused on activity-specific injuries by comparing injury risks within specific sports or activities [11,35-37]. However, in this population-based study PA was not among the factors that explain unintentional injuries, even in the univariate analysis. The present findings seem to be consistent with other research, which found no association between leisure-time physical activity and overall injuries [35]. The risk of activity-related injury has shown to be greater for physically active people, whereas the risk of other types of injuries has shown to be greater for sedentary people [35]. Perhaps different results would be obtained if we had studied a younger population whose physical activity behaviors would likely have been different. Furthermore, there was no difference in the prevalence of unintentional injuries in the high, moderate, or low PA categories in groups according DS.

On the other hand, PA could be beneficial with respect to injury prevention, as it has proven to be strictly related to functional ability [12,13]. PA contributes favorably to balance, muscle strength, and neuromuscular control [32], which in turn improves functional ability. A recently published injury prevention report encourages adults to maintain and increase their sporting and physical activity behaviors and suggests taking up activities appropriate for their age and individual level of fitness and experience [38]. Furthermore, the report emphasizes the importance of sufficient PA among elderly people for maintaining independency and reducing falls and fractures [38].

In the present study, those with DS had higher amounts of activity loss days after unintentional injuries. Wan et al. also reported longer lengths of hospital stay in mentally ill participants after unintentional injury [39]. One explanation behind this longer recovery period might be that the presence of DS affects the perceived disability by lowering the sense of complete recovery after the injury [4]. Another explanation may be the lower functional ability among those with DS reported in this study because functional ability has proved to be highly predictive for recovery after injury [40].

Some limitations have to be acknowledged in this study. Due to participation rate of 64% in this study with a random sample of the population, we cannot exclude the effect of selection bias on the results. On the other hand, those who did not participate were younger, and thus cannot directly be assumed that they have worse overall health status. Further females who are stated to have higher prevalence of DS were more likely to participate in the study than males. The information about injuries was collected retrospectively; as a result, participants do not necessarily remember all the injuries they had during the previous 12 months. People tend to forget about 30% of their injuries over the course of a year [41]. However, because we limited the unintentional injuries only to those needing medical attention, the possibility of this source of error becomes minimal. The assessment of DS and total PA relied on self-reported questionnaires, which have the potential for error in judgment, recall difficulties, misinterpretation of questions, and to elicit socially desirable responses. However, both the BDI and IPAQ questionnaires are validated and widely used in these areas, which also allows the comparison of the results with other studies [21,42]. The main strength is the large population-based data on males and females. However, because of a lower age limit of 45 years in this study, the results cannot be generalized to younger individuals. Further, the present cross-sectional design does not allow drawing of a causal conclusion. Longitudinal studies should be done to assess the prospective relationships between PA, DS and unintentional injuries.

## Conclusions

The main result of this study showed that depressive symptoms were related to a higher prevalence of unintentional injuries and prolonged activity-loss after injury. In addition, lower functional ability and musculoskeletal diseases were significantly related to the occurrence of unintentional injuries, whereas physical activity level was not. These results underline the importance of injury prevention, particularly among those who have DS and additional risk factors by increasing safety at home and during leisure-time activities.

## Abbreviations

PA: Physical activity; DS: Depressive symptoms; FIN-D2D: The Finnish type 2 diabetes survey; MONICA: Multinational monitoring of trends and determinants in cardiovascular disease; BMI: Body mass index; IPAQ: International Physical Activity Questionnaire; MET: Metabolic equivalent; BDI: Beck depression inventory; SD: Standard deviations; CI: Confidence intervals.

## Competing interests

Dr. Kampman has been acting as a consulting expert for Janssen, Pfizer and Lundbeck. All other authors declare that they have no conflicts of interest to report.

## Authors’ contributions

KK, AH, HJK, and MV participated in designing the study and drafted the manuscript. HK and SJ participated in designing the study, performed the statistical analysis and drafted the manuscript. All authors have made substantial contributions to conception and design, or acquisition of the data, participated in drafting of the article or critical revision for important intellectual content. All authors read and approved the final manuscript.

## Pre-publication history

The pre-publication history for this paper can be accessed here:

http://www.biomedcentral.com/1471-2458/12/516/prepub
